# Focusing field epidemiology training on national health priorities in Papua New Guinea: consultative prioritization, from health workers to policy-makers

**DOI:** 10.5365/wpsar.2025.16.1.1105

**Published:** 2025-03-24

**Authors:** Tambri Housen, Barry Ropa, James Flint, Tony Merritt, Rachel Hammersley-Mather, Alois Pukienei, Rosheila Dagina, Bethseba Peni, Martha Pogo, David N Durrheim

**Affiliations:** a School of Medicine and Public Health, University of Newcastle, Newcastle, New South Wales, Australia.; b National Department of Health, Port Moresby, Papua New Guinea.; c Hunter New England Population Health Unit, Newcastle, New South Wales, Australia.; d Health Authority, Buka, Autonomous Region of Bougainville, Papua New Guinea.; e Provincial Health Authority, Kimbe, West New Britain, Papua New Guinea.

Papua New Guinea (PNG) faces significant public health threats: low immunization coverage; weak primary health-care systems; high maternal mortality; repeated outbreaks of circulating vaccine-derived poliovirus type 1, measles, cholera, dengue and chikungunya; uncontrolled multidrug-resistant tuberculosis; and the emergence of extensively drug-resistant tuberculosis, Zika virus disease and Japanese encephalitis. The generation of high-quality, policy-relevant knowledge is critical to enable evidence-informed decisions that will strengthen PNG’s health systems and effectively manage health threats. PNG’s 2012 guide to health research policy identified a need for research targeting national health priorities. (*1*) In 2018, we conducted a prioritization exercise to identify key prioritization areas (KPAs) for operational research projects to be undertaken by fellows completing a new, advanced Field Epidemiology Training Programme in PNG (aFETPNG) during 2019–2021. The aFETPNG programme aimed to build evidence to inform policy and practice, and focus on strengthening health systems in PNG.

## Methods

**Fig. 1:**
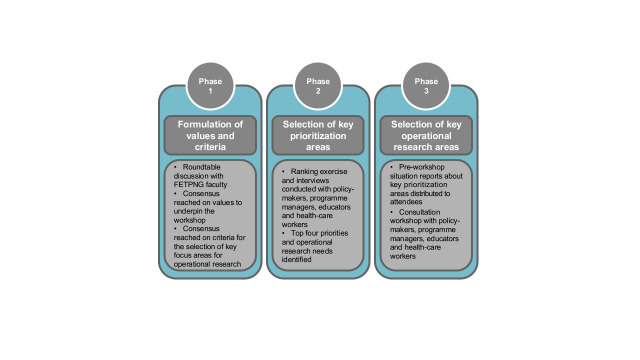
Process for selecting operational research priorities for the advanced Field Epidemiology Training Program in Papua New Guinea (aFETPNG), 2018

The prioritization exercise occurred during October–November 2018. Several health research priority setting methodologies were reviewed to identify a systematic approach suited for adaptation to our needs. (*2*-*6*) Our methods synthesized elements of all reviewed approaches, and adapted Viergever et al.’s checklist for health research priority setting. (*7*) **Fig. 1** illustrates the three-phase approach adopted in this prioritization exercise.

[**insert** Figure 1]

The initial list of health priorities for ranking was drawn from the PNG National Health Plan 2011–2020, (*8*) the Papua New Guinea–WHO Country Cooperation Strategy 2016–2020, (*9*) the Asia Pacific Strategy for Emerging Diseases and Public Health Emergencies III (*10*) and the PNG International Health Regulations Core Capacity Development Plan 2014–2016. (*11*)

In Phase 1, faculty from the FETPNG met to discuss and agree on key values to underpin the prioritization process; the nominal group technique (*12*) was used to gain consensus. During the same meeting, the faculty identified and finalized criteria for the prioritization of focus areas for operational research. For this study, we defined operational research as research that examines factors associated with the implementation of programmatic activities. Operational research questions are targeted at identifying and addressing factors that have a direct impact on the quality and effectiveness of the delivery of health services.

During Phase 2 of the prioritization process, 39 stakeholder representatives were identified and engaged from various departments and organizations, including the National Department of Health, provincial health authorities, district health authorities, programme management, the health-care workforce, the World Health Organization, the University of Papua New Guinea, church-run health services, the United Nations Population Fund, Pacific Adventist University, the National Agriculture Quarantine and Inspection Authority and FETPNG.

These representatives were given a questionnaire to rank 14 priority areas for operational research based on perceived public health importance, with 1 being the most important and 14 the least.

For each respondent, the four highest ranked priorities were weighted in the following way: areas ranked as priority 1 were given a score of 4; those ranked as priority 2 were given a score of 3; priority 3 was given a score of 2; and priority 4 was given a score of 1.

To explore the reasons why certain areas were prioritized, 18 of the stakeholder representatives were invited to participate in semi-structured interviews. Participants were selected based on efforts to include a diversity of expertise, gender and region, as well as availability. The interviews explored perceptions about the reasons why a KPA was chosen, what was currently working well in that area, operational research needs, potential barriers to conducting operational research, the potential for policy and programmatic changes, and the proposed beneficiaries of research outputs. Interviews were recorded, transcribed and analysed using NVivo software (version 11, Lumivero, Denver, CO, USA). Structural codes of segments of text were created and categorized into broader subcategories, which were then collated by KPA under overarching themes.

Integrated analysis of the data collected during Phases 1 and 2 informed the design of a consultation workshop (Phase 3). A situation report for each KPA was compiled that included information about the burden of disease, current knowledge, recent developments, current policies, future focus and alignment with the National Health Plan. (*8*) These were circulated to invited workshop participants and were available during the workshop for further review. Invitees included policy-makers, programme managers, educators and health-care workers.

During the consultation workshop on 24 November 2018, programme managers for the identified KPAs provided a brief overview of the context and key challenges associated with meeting programmatic targets. Participants brainstormed key operational research areas (KORAs) before grouping them into overarching themes: supply, procurement and distribution, governance, workforce, quality of care, service delivery, data management, health-related behaviour and access to services.

The KORAs were used to direct the formulation of operational research questions. Questions were reviewed against previously developed assessment criteria, and those meeting the criteria were ranked using consensus ranking.

The workshop concluded with an overall evaluation of the prioritization process. This evaluation was guided by six questions addressing each workshop activity and participants’ perceptions of the overall utility of the exercise.

## Results

In Phase 1, eight FETPNG faculty agreed on four values to underpin the prioritization process: operational research should improve current health systems (8/8), have the potential to reduce mortality and morbidity (7/8), contribute to policy and practice (7/8) and contribute to evidence (4/8).

Consensus was reached on seven criteria for the selection of key focus areas, with three identified as mandatory: the operational research must be ethical, implementable using existing resources, and able to be completed within an 18-month time frame. Consideration of four additional criteria was deemed non-mandatory but important: the magnitude of the health problem (8/8); demonstrated effectiveness, i.e. the potential for the proposed research to address objectives (5/8); the potential for recommendations to be successfully implemented (5/8); and the size of the knowledge gap or lack of adequate implementation (4/8).

All 39 identified stakeholders completed the ranking exercise (100% response rate). **Table 1**) provides the results of the prioritization exercise; the top four KPAs identified were: vaccine-preventable diseases and immunization, health systems strengthening, maternal and reproductive health, and communicable disease control.

**Table 1:** Weighted prioritization of key priority areas for operational research to be conducted by fellows of the advanced Field Epidemiology Training Programme in Papua New Guinea, 2018^a^

Key areas for prioritation	Priority weighting				Total
	1	2	3	4	
**Vaccine-preventable diseases and immunization**	**40**	**24**	**12**	**2**	**78**
**Health systems strengthening**	**56**	**6**	**6**	**4**	**72**
**Maternal and reproductive health**	**12**	**30**	**12**	**7**	**61**
**Communicable disease control**	**20**	**18**	**12**	**5**	**55**
**Child health**	**12**	**15**	**16**	**3**	**46**
**Public health emergency preparedness**	**4**	**6**	**6**	**7**	**23**
**Zoonotic diseases**	**8**	**3**	**0**	**0**	**11**
**Laboratory capacity**	**0**	**3**	**4**	**2**	**9**
**Vector-borne diseases**	**0**	**0**	**2**	**4**	**6**
**Healthy lifestyles**	**0**	**3**	**0**	**1**	**4**
**Infection prevention and control**	**0**	**0**	**2**	**1**	**3**
**Noncommunicable diseases**	**0**	**0**	**2**	**0**	**2**
**Access to medical products**	**0**	**0**	**0**	**0**	**0**
**Diarrhoeal disease**	**0**	**0**	**0**	**0**	**0**

^a^ Key priority areas were weighted in the following way: those rated as priority 1 were given a score of 4; those rated as priority 2 were given a score of 3; priority 3 was given a score of 2; and priority 4 was given a score of 1.

All 18 individuals invited for interview agreed (100% response rate). Key themes emerging from the interviews encompassed challenges related to governance, workforce capacity, data collection, management and reporting, as well as logistics, including resourcing, supply, procurement and distribution. Additional themes highlighted issues around access to health services; health-seeking behaviour; knowledge, attitudes and practices; service delivery; and the quality of care.

Twenty-one participants attended the consultation workshop, including clinicians, clinical managers in health facilities, district and provincial health staff, and programme managers from the National Department of Health.

Sixteen operational research questions were developed under KPA1 (vaccine-preventable diseases and immunization), 16 under KPA2 (health systems strengthening) and 19 under KPA3 (maternal and reproductive health). Due to time constraints, questions were not developed for KPA4 (communicable disease control); these were developed later by aFETPNG fellows in consultation with national programme managers. Research questions for each KPA, grouped by the KORA, are available in **Supplementary Table 1**.



All participants completed the post-workshop evaluation. Participants felt that the prioritization exercise provided a transparent and collaborative approach to reaching collective decisions about focus areas for operational research. The involvement of a cross-section of stakeholders from each tier of the health system was viewed as a strength. For example, one participant commented that, “The workshop was transparent. Many people come and say this is what we have developed, but in this process, we were engaged; it is our contribution.”

## Discussion

Building on the success of the intermediate FETPNG, the aFETPNG took a more systematic approach to aligning fellows’ projects with national health priorities. The prioritization exercise focused on strengthening health systems by building a body of evidence around identified KPAs: vaccine-preventable diseases and immunization, health systems strengthening, maternal and reproductive health, and communicable disease control. Altogether, 17 operational research projects were conducted during 2019–2021 by fellows of aFETPNG with support from the Global Outbreak Alert and Response Network (GOARN), which provided technical and logistical support through the University of Newcastle, Australia. During this period, COVID-19 was added as a fifth KPA.

The methodology described in this report provides a model for aligning field epidemiology fellows’ projects, and operational research more generally, with national priority areas. The inclusive and transparent approach fostered ownership of identified priorities by those involved in the process, increasing the likelihood of translational impact. The process also strengthened links among stakeholders across the health sector and fostered greater understanding and appreciation for others’ roles, accountabilities and challenges. Key lessons learned were the importance of including national programme managers in formulating KORAs and questions. The national managers provided invaluable context in discussions, highlighting gaps in knowledge and evidence for policy development. The small sample size may have led to biased results; however, the broad representation of stakeholders provided the opportunity to capture diverse views. This approach could be adopted by other GOARN partners and relevant stakeholders, who aim to support research prioritization for operational research, to ensure such research is driven by national priorities.
