# Supplementary material for: Focusing field epidemiology training on national health priorities in Papua New Guinea: consultative prioritization, from health workers to policy-makers
Source: Western Pac Surveill Response J. 2025 Mar 24;16(1):1–5. doi: 10.5365/wpsar.2025.16.1.1105 (PMC11937779; doi:10.5365/wpsar.2025.16.1.1105)
Supplement: Supplementary file 1 [file wpsar-16-1105-s001.pdf]

Supplementary Table 1. **Operational research questions by key operational research area for the advanced Field Epidemiology Training Programme, Papua New Guinea, 2018**

Key operational research area	Operational research question
**KPA1: vaccine-preventable diseases and immunization[a]**	
Governance	*What factors provided by the field epidemiology training programme would enhance delivery at the provincial and district levels?
Quality of care	What are the minimum required services for routine immunization? Are these being implemented?
Workforce	How do supervision visits impact the motivation of the workforce to improve service delivery?
	*What are the key challenges for health workers in providing a routine immunization service?
	What are the core competencies for health workers related to VPDs and immunization? What training do health workers receive on VPDs and immunization, and is this directly related to the core competencies?
Supply, procurement and distribution	*What are the key resources required to deliver routine immunizations?
	How many vaccine fridges are functioning across the provinces? Which power sources are most reliable?
	What are the impacts of supply, procurement and distribution on routine immunizations?
Access	What are the barriers to accessing immunization? What impact do these barriers have on coverage?
	What are the factors contributing to poor access to health facilities?
	Do mobile clinics improve the uptake of immunization?
Data management	*How effective is the current vaccination data collection and reporting system?
	What are the factors contributing to underreporting of vaccination status? Is underreporting due to a lack of knowledge among health service workers?
	Are health centre staff conducting active surveillance after notification of a VPD?
Health-related behaviours	*What are the knowledge, attitudes and practices related to the utilization of child health registers in informing vaccination activities?
	*What are health-care workers' knowledge, attitudes and practices related to surveillance for VPDs and immunization?
**KPA2: health systems strengthening[a]**	
Access	*What are the barriers to and enablers of community access to care at health services and aid posts?
	*What are the barriers to and enablers of health-care workers conducting outreach services?
Workforce	*How does improving the skills of village birth attendants and village health volunteers lead to increased access to integrated care? What are the barriers to and required incentives for enhancing engagement of village birth attendants and village health volunteers with health-care workers?
	*What factors are affecting staff recruitment and retention in rural areas?
	What different types of training programmes are available to health staff? How can multiskilled training approaches strengthen primary health care?
	How do staff attitudes towards people presenting at health facilities affect the utilization of health services?
	What is the current health workforce capacity in public health domains? What are the training needs of the health workforce?
	What are the gaps in knowledge among health-centre staff that need filling to deliver an integrated approach to primary health care?
	What different types of training programmes are available to health staff? How can multiskilled training approaches strengthen primary health care?
	Does empowering village health volunteers have an impact on health services in the community? What skills do village health volunteers need?
Health-related behaviours	*How do men act as barriers to and facilitators of access to health care? How can they be engaged to act as partners in health care?
	How knowledgeable is the community about health-related issues and the available services?

Key operational research area	Operational research question
Service delivery	*What is the impact of the health island concept on health-care outcomes and service delivery?
	What strategies can be developed to review, integrate and improve current outreach services without negatively impacting existing health services?
Quality of care	Do village health volunteers give medication in accordance with national guidelines?
**KPA3: maternal and reproductive health[a]**	
Health-related behaviours	*What are the roles of men in enabling and preventing the uptake of reproductive health services? How do they see themselves participating more?
	*What are the contributing factors to adolescent pregnancy?
	*Which sexual and reproductive health topics are taught in schools? How effective are current teaching methods?
	What influences do religion and tradition have on the use of contraception? Which are the most prescribed contraceptives?
	What are the knowledge, attitudes and practices of teachers delivering sexual and reproductive health education in schools?
	What are the enabling factors associated with health-seeking behaviours for sexual and reproductive health?
Service delivery	*Which factors are the most important to shortening the three delays?[c]
	*What are the barriers to accessing antenatal care? How can current outreach services be reviewed and optimized through new strategies to integrate and improve them without affecting existing health services?
	Which demographic factors are associated with the uptake of family planning?
	What options are available to terminate a pregnancy? What are the most common pathways?
Quality of care	How do unsupervised, community-based delivery outcomes compare with delivery outcomes at health centres?
	What is the association between the age of a mother and complications during delivery?
	What is the level of knowledge among health-care workers regarding the use of a cervicograph and referral practices?
	Are guidelines being followed appropriately during delivery at health-care centres?
	What are the key indicators of high-quality antenatal care, and how effectively are they being implemented and measured in clinical practice?
Supply, procurement and distribution	What are the most common family planning commodities available, and how frequently do they run out? What commodities are unavailable? What are the most popular commodities?
	At what point in the supply chain does distribution of family planning fail?
Access	*What are the barriers preventing access to family planning?
	What proportion of antenatal visits are made by pregnant teenagers?
**KRA4: communicable disease control[b]**	
Health-related behaviours	What cultural and geographical factors affect the transmission of diseases within a population?
	What is the social impact of TB infection on patients and their families?
	What are the effects of self-stigma on individuals with TB infection?
Data management	*What is the prevalence of drug-sensitive TB compared with multidrug-resistant TB in Papua New Guinea?
	*What is the prevalence of TB in high-risk populations, and what are the current management practices for these groups?
	*What strategies can be implemented to improve data collection and enhance the timeliness of TB reporting?
Service delivery	What are the most effective methods for administering prophylaxis to contacts of individuals with TB?
	*What is the prevalence of TB among school-aged children?

Key operational research area	Operational research question
Governance	What are the roles of provincial and district-level health authorities in providing care and treatment for individuals with TB?
	How do nongovernmental partners contribute to providing care and treatment for individuals with TB?
	What is the financial impact of TB treatment on patients and their families?
Workforce	How do non-monetary incentives and upskilling initiatives influence the retention and effectiveness of voluntary treatment partners in TB care?
	What strategies can effectively identify and address the factors contributing to high staff turnover in TB care and treatment programmes?
Quality of care	*Which interventions are most effective in reducing loss to follow-up and ensuring optimal case management for individuals with TB?
	*Which strategies can enhance the identification and treatment of individuals with HIV/TB coinfection?
	How do current TB diagnostic practices contribute to delayed diagnosis, and what improvements can be made to expedite detection?
	What impact do waiting times at health centres have on the quality of care provided to patients?

KPA: key prioritization area; TB: tuberculosis; VPD: vaccine-preventable disease.

[a] Group consensus about ranking was reached for KPA1–KPA3; the top six priorities are marked with an *.

[b] Due to time constraints, operational research questions were not developed for KPA4. The key operational research areas identified during the brainstorming session were ranked by group consensus. The top six priorities are marked with an *.

[c] The three delays in maternal and reproductive health care refer to (1) delay in the decision to seek care, (2) delay in reaching care and (3) delay in receiving adequate care.
